# Supplementary material for: Maternal Biomarkers of Acetaminophen Use and Offspring Attention Deficit Hyperactivity Disorder
Source: Brain Sci. 2018 Jul 3;8(7):127. doi: 10.3390/brainsci8070127 (PMC6071105; doi:10.3390/brainsci8070127)
Supplement: Supplementary file 1 [file brainsci-08-00127-s001.pdf]

Figure S1. Flowchart of sample included in the analysis.

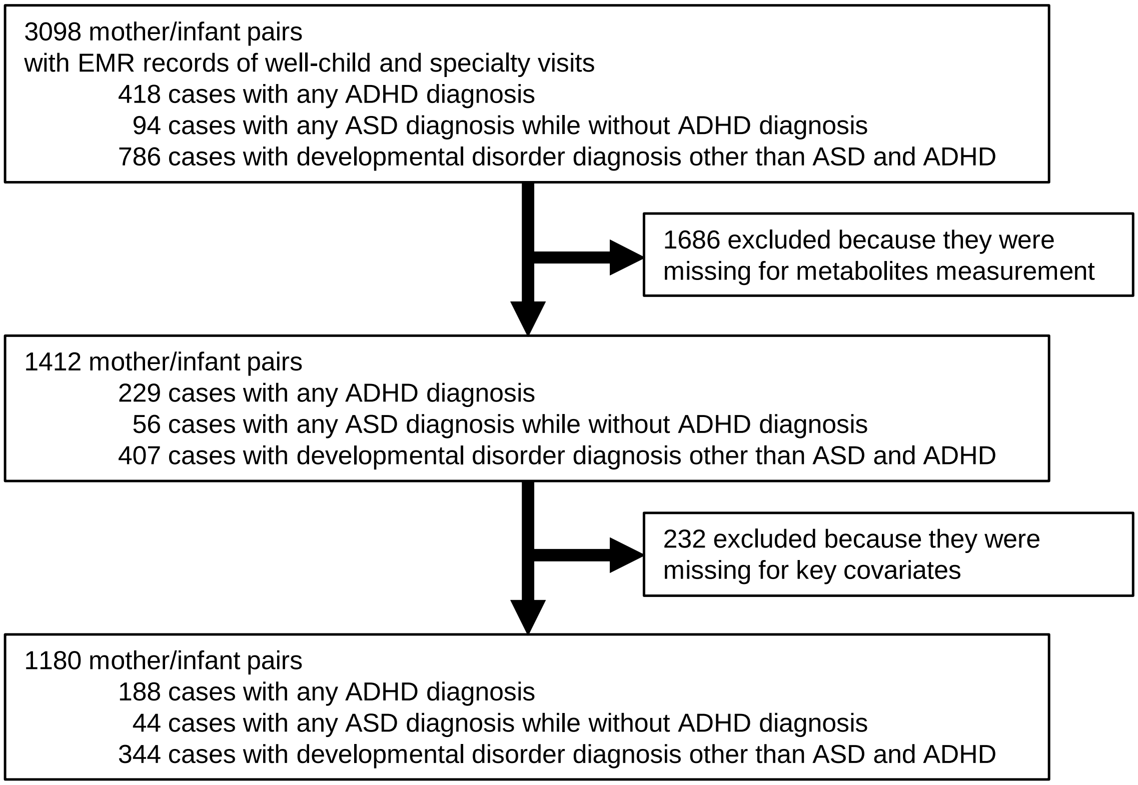

Table S1. Maternal and child characteristics for participants excluded and included in the analysis.

Variable	Total, N (%)	Excluded, N (%)	Included, N (%)	P-value‡
Total	3098 (100)	1918 (61.91)	1180 (38.09)	
Maternal Age				0.796
<35	2534 (81.79)	1569 (81.80)	965 (81.78)	
≥35	556 (17.95)	341 (17.78)	215 (18.22)	
Education level				0.109
Below college degree	2642 (85.28)	1609 (83.89)	1033 (87.54)	
College degree or above	420 (13.56)	273 (14.23)	147 (12.46)	
Maternal race/ethnicity				<0.001
Black	1965 (63.43)	1156 (60.27)	809 (68.56)	
White	227 (7.33)	179 (9.33)	48 (4.07)	
Hispanic	682 (22.01)	426 (22.21)	256 (21.69)	
Other	209 (6.75)	142 (7.40)	67 (5.68)	
Smoking before or during pregnancy				0.222
Never	2496 (80.57)	1519 (79.20)	977 (82.80)	
Quitter	238 (7.68)	148 (7.72)	90 (7.63)	
Continuous	330 (10.65)	217 (11.31)	113 (9.58)	
Drinking before or during pregnancy				0.627
No	2740 (88.44)	1654 (86.24)	1086 (92.03)	
Yes	247 (7.97)	153 (7.98)	94 (7.97)	
Child's sex				0.617
Female	1529 (49.35)	953 (49.69)	576 (48.81)	
Male	1567 (50.58)	963 (50.21)	604 (51.19)	
Delivery type				0.930
C-section	1116 (36.02)	690 (35.97)	426 (36.10)	
Vaginal	1967 (63.49)	1213 (63.24)	754 (63.90)	
Gestational age, week				0.003
Mean (SD)	37.6(3.5)	37.5(3.6)	37.9(3.3)	
Birthweight, g				<0.001
Mean (SD)	2898.3(819.7)	2856.5(834.9)	2966.2(789.9)	

Note: ‡The p-values were obtained from χ^2 tests or t-tests between children included in and excluded from the main analysis.

Table S2. List of ICD-9 and ICD-10 codes for the diagnosis of each developmental disorder.

Developmental disorder	ICD-9 codes	ICD-10 codes
ASD	299.0, 299.00, 299.01, 299.8, 299.80, 299.81, 299.9, 299.90, 299.91	F84.0, F84.8, F84.9
ADHD	314.0, 314.00, 314.01, 314.1, 314.2, 314.8, 314.9	F90, F90.0, F90.1, F90.2, F90.8, F90.9
Developmental delays	315.0-315.9	F81.0, R48.0, F81.81, F81.2, F81.89, F80.1, F80.2, H93.25, F80.4, F80.81, F80.0, F80.82, F80.89, F82, F88, F81.9, F89
Intellectual disabilities	317-319	F70, F71, F72, F73, F78, F79

Table S3. The association between maternal acetaminophen metabolites and the risk of ADHD diagnosis only, ASD diagnosis only, and diagnoses of both ADHD and ASD in offspring.

Model		ADHD only, N=166			ASD only, N=44			ADHD and ASD, N=22		
		Odds Ratio	95% CI	P-value	Odds Ratio	95% CI	P-value	Odds Ratio	95% CI	P-value
Unchanged acetaminophen*										
Model 1	Second tertile	1.92	(1.22,3.01)	0.005	1.09	(0.49,2.45)	0.832	0.79	(0.28,2.25)	0.657
	Third tertile	2.29	(1.47,3.57)	<0.001	1.79	(0.86,3.73)	0.118	0.95	(0.35,2.61)	0.928
Model 2	Second tertile	1.96	(1.20,3.20)	0.007	0.96	(0.41,2.25)	0.932	0.85	(0.28,2.64)	0.785
	Third tertile	2.32	(1.38,3.89)	0.001	1.32	(0.57,3.07)	0.523	0.92	(0.27,3.20)	0.901
Model 3	Second tertile	1.96	(1.20,3.20)	0.007	0.97	(0.41,2.28)	0.947	0.91	(0.29,2.83)	0.868
	Third tertile	2.32	(1.38,3.89)	0.001	1.32	(0.57,3.08)	0.516	0.92	(0.26,3.21)	0.891
Model 4	Second tertile	1.95	(1.19,3.18)	0.008	0.96	(0.41,2.25)	0.926	0.85	(0.27,2.63)	0.778
	Third tertile	2.30	(1.37,3.86)	0.002	1.31	(0.56,3.05)	0.534	0.91	(0.26,3.14)	0.884
Model 5	Second tertile	1.97	(1.21,3.22)	0.007	0.96	(0.41,2.24)	0.920	0.85	(0.28,2.64)	0.783
	Third tertile	2.29	(1.37,3.85)	0.002	1.33	(0.57,3.10)	0.509	0.93	(0.27,3.24)	0.912
Model 6	Second tertile	2.01	(1.22,3.29)	0.006	0.98	(0.42,2.29)	0.958	0.91	(0.29,2.85)	0.872
	Third tertile	2.28	(1.36,3.84)	0.002	1.33	(0.57,3.10)	0.516	0.92	(0.26,3.22)	0.892
3-(N-Acetyl-L-cystein-S-yl) acetaminophen*										
Model 1	Below median	1.82	(1.17,2.83)	0.008	0.91	(0.40,2.07)	0.819	0.91	(0.32,2.60)	0.858
	Above median	2.31	(1.50,3.55)	<0.001	1.81	(0.89,3.65)	0.100	1.11	(0.41,3.03)	0.841
Model 2	Below median	1.90	(1.18,3.05)	0.008	0.77	(0.33,1.82)	0.557	0.98	(0.32,2.99)	0.974
	Above median	2.28	(1.37,3.80)	0.002	1.19	(0.52,2.73)	0.688	0.90	(0.26,3.13)	0.868
Model 3	Below median	1.91	(1.18,3.08)	0.008	0.78	(0.33,1.86)	0.576	1.12	(0.37,3.43)	0.843
	Above median	2.28	(1.37,3.81)	0.002	1.19	(0.52,2.75)	0.680	0.91	(0.26,3.18)	0.888
Model 4	Below median	1.87	(1.16,3.01)	0.010	0.76	(0.32,1.80)	0.537	1.00	(0.33,3.07)	0.996
	Above median	2.25	(1.34,3.76)	0.002	1.17	(0.51,2.70)	0.717	0.89	(0.26,3.08)	0.860
Model 5	Below median	1.88	(1.17,3.03)	0.009	0.78	(0.33,1.85)	0.576	1.00	(0.33,3.05)	0.994
	Above median	2.25	(1.35,3.75)	0.002	1.21	(0.52,2.78)	0.660	0.91	(0.26,3.18)	0.882
Model 6	Below median	1.93	(1.19,3.11)	0.008	0.80	(0.33,1.89)	0.604	1.15	(0.37,3.53)	0.812
	Above median	2.24	(1.34,3.76)	0.002	1.20	(0.52,2.78)	0.676	0.91	(0.26,3.20)	0.889
Acetaminophen glucuronide*										
Model 1	Below median	1.69	(1.09,2.60)	0.018	1.97	(0.94,4.13)	0.074	0.66	(0.21,2.07)	0.471
	Above median	2.16	(1.44,3.26)	<0.001	1.82	(0.86,3.88)	0.118	0.98	(0.36,2.65)	0.964
Model 2	Below median	1.67	(1.04,2.68)	0.033	1.39	(0.63,3.07)	0.411	0.51	(0.15,1.79)	0.293
	Above median	2.30	(1.40,3.78)	0.001	1.20	(0.50,2.88)	0.680	0.88	(0.24,3.20)	0.845
Model 3	Below median	1.67	(1.04,2.69)	0.034	1.42	(0.64,3.15)	0.390	0.56	(0.16,2.00)	0.373
	Above median	2.30	(1.40,3.78)	0.001	1.22	(0.51,2.92)	0.663	0.88	(0.24,3.22)	0.851
Model 4	Below median	1.66	(1.04,2.66)	0.035	1.38	(0.63,3.05)	0.422	0.51	(0.14,1.79)	0.293
	Above median	2.26	(1.37,3.72)	0.001	1.19	(0.49,2.86)	0.702	0.88	(0.24,3.17)	0.845
Model 5	Below median	1.68	(1.05,2.69)	0.032	1.39	(0.63,3.06)	0.418	0.51	(0.15,1.78)	0.290
	Above median	2.28	(1.38,3.75)	0.001	1.21	(0.50,2.91)	0.668	0.89	(0.24,3.25)	0.858
Model 6	Below median	1.74	(1.08,2.81)	0.023	1.44	(0.65,3.20)	0.373	0.56	(0.16,2.02)	0.378
	Above median	2.26	(1.37,3.73)	0.001	1.21	(0.50,2.92)	0.675	0.88	(0.24,3.24)	0.852

Model		ADHD only, N=166			ASD only, N=44			ADHD and ASD, N=22		
		Odds Ratio	95% CI	P-value	Odds Ratio	95% CI	P-value	Odds Ratio	95% CI	P-value
Acetaminophen burden**										
Model 1	Below median	1.77	(1.15,2.71)	0.009	1.85	(0.87,3.93)	0.110	0.66	(0.21,2.08)	0.478
	Above median	2.08	(1.38,3.14)	0.001	1.94	(0.92,4.08)	0.080	0.97	(0.36,2.64)	0.954
Model 2	Below median	1.76	(1.10,2.80)	0.018	1.32	(0.59,2.95)	0.495	0.52	(0.15,1.81)	0.303
	Above median	2.16	(1.31,3.55)	0.003	1.30	(0.55,3.08)	0.553	0.86	(0.24,3.12)	0.819
Model 3	Below median	1.76	(1.10,2.82)	0.019	1.35	(0.60,3.02)	0.471	0.57	(0.16,2.03)	0.387
	Above median	2.16	(1.31,3.55)	0.003	1.31	(0.55,3.12)	0.539	0.86	(0.24,3.14)	0.822
Model 4	Below median	1.74	(1.09,2.79)	0.020	1.31	(0.59,2.93)	0.506	0.52	(0.15,1.82)	0.303
	Above median	2.12	(1.28,3.50)	0.003	1.28	(0.54,3.06)	0.572	0.86	(0.24,3.09)	0.819
Model 5	Below median	1.77	(1.11,2.82)	0.017	1.31	(0.59,2.93)	0.505	0.52	(0.15,1.80)	0.299
	Above median	2.13	(1.29,3.52)	0.003	1.31	(0.55,3.12)	0.539	0.87	(0.24,3.17)	0.832
Model 6	Below median	1.84	(1.14,2.96)	0.012	1.37	(0.61,3.07)	0.452	0.57	(0.16,2.05)	0.392
	Above median	2.11	(1.27,3.49)	0.004	1.30	(0.55,3.12)	0.550	0.86	(0.24,3.15)	0.823

Note: NT was defined as free of any developmental disorder diagnosis; ADHD only was defined as any ADHD diagnosis without having an ASD diagnosis; ASD only was defined as any ASD diagnosis without having an ADHD diagnosis; ADHD and ASD was defined as having both ADHD and ASD diagnosis;
Model 1: Multinomial logistic regression without adjustment;
Model 2: Model 1 further adjusted for maternal age at delivery, maternal race/ethnicity, maternal education, smoking before or during pregnancy, drinking before or during pregnancy, maternal BMI, parity, child's sex, delivery type, preterm birth, and birthweight;
Model 3: Model 2 further adjusted for maternal fever during pregnancy;
Model 4: Model 2 further adjusted for maternal intrauterine infection/inflammation during pregnancy;
Model 5: Model 2 further adjusted for breastfeeding;
Model 6: Model 2 further adjusted for maternal fever, maternal intrauterine infection/inflammation during pregnancy, and breastfeeding.
* Inverse normal transformed intensity ** Sum of all the acetaminophen metabolites.
Unchanged acetaminophen: first tertile as reference; For other exposures: no detection as reference

Table S4. The association between maternal acetaminophen metabolites and the risk of ADHD diagnosis, ASD diagnosis (excluding ADHD), and other DD diagnosis in offspring using propensity score weighted multinomial logistic regression models.

Model		ADHD,188(15.9%)			ASD,44(3.7%)			otherDD,344(29.2%)		
		Odds Ratio	95% CI	P-value	Odds Ratio	95% CI	P-value	Odds Ratio	95% CI	P-value
Unchanged acetaminophen*										
Model 1	Second tertile	2.48	(1.56,3.96)	<0.001	1.14	(0.56,2.32)	0.712	0.94	(0.69,1.26)	0.665
	Third tertile	3.75	(2.38,5.89)	<0.001	1.67	(0.85,3.26)	0.136	1.10	(0.81,1.48)	0.547
Model 2	Second tertile	3.02	(1.83,4.97)	<0.001	1.58	(0.74,3.35)	0.238	1.16	(0.83,1.60)	0.389
	Third tertile	4.14	(2.56,6.71)	<0.001	1.72	(0.85,3.47)	0.130	1.05	(0.76,1.46)	0.770
Model 3	Second tertile	3.05	(1.85,5.03)	<0.001	1.68	(0.78,3.60)	0.183	1.13	(0.81,1.58)	0.456
	Third tertile	4.14	(2.55,6.70)	<0.001	1.79	(0.88,3.61)	0.106	1.05	(0.75,1.45)	0.790
Model 4	Second tertile	2.99	(1.81,4.94)	<0.001	1.64	(0.77,3.52)	0.201	1.14	(0.82,1.59)	0.421
	Third tertile	4.12	(2.54,6.67)	<0.001	1.80	(0.89,3.64)	0.103	1.04	(0.75,1.45)	0.804
Model 5	Second tertile	2.98	(1.80,4.91)	<0.001	1.57	(0.74,3.34)	0.240	1.15	(0.83,1.60)	0.401
	Third tertile	4.05	(2.50,6.57)	<0.001	1.71	(0.85,3.46)	0.134	1.04	(0.75,1.44)	0.824
Model 6	Second tertile	3.00	(1.81,4.95)	<0.001	1.68	(0.78,3.60)	0.183	1.13	(0.81,1.57)	0.467
	Third tertile	3.96	(2.44,6.44)	<0.001	1.78	(0.87,3.61)	0.112	1.03	(0.74,1.43)	0.857
3-(N-Acetyl-L-cystein-S-yl) acetaminophen*										
Model 1	Below median	2.98	(1.87,4.77)	<0.001	1.85	(0.91,3.75)	0.088	2.06	(1.53,2.76)	<0.001
	Above median	4.35	(2.75,6.88)	<0.001	2.28	(1.13,4.60)	0.021	1.65	(1.20,2.27)	0.002
Model 2	Below median	3.45	(2.11,5.64)	<0.001	2.19	(1.05,4.55)	0.037	2.19	(1.59,3.02)	<0.001
	Above median	4.60	(2.81,7.54)	<0.001	2.20	(1.05,4.62)	0.037	1.44	(1.02,2.03)	0.041
Model 3	Below median	3.52	(2.15,5.78)	<0.001	2.38	(1.14,4.97)	0.022	2.18	(1.58,3.01)	<0.001
	Above median	4.61	(2.82,7.55)	<0.001	2.32	(1.11,4.87)	0.026	1.43	(1.01,2.03)	0.043
Model 4	Below median	3.43	(2.09,5.61)	<0.001	2.30	(1.10,4.82)	0.027	2.18	(1.58,3.01)	<0.001
	Above median	4.58	(2.79,7.50)	<0.001	2.34	(1.11,4.92)	0.025	1.43	(1.01,2.03)	0.044
Model 5	Below median	3.38	(2.06,5.54)	<0.001	2.19	(1.05,4.56)	0.037	2.17	(1.58,2.99)	<0.001
	Above median	4.50	(2.75,7.38)	<0.001	2.20	(1.05,4.61)	0.037	1.42	(1.00,2.01)	0.047
Model 6	Below median	3.46	(2.11,5.69)	<0.001	2.39	(1.14,5.00)	0.021	2.17	(1.57,2.99)	<0.001
	Above median	4.41	(2.68,7.25)	<0.001	2.31	(1.10,4.88)	0.028	1.41	(1.00,2.01)	0.052
Acetaminophen glucuronide*										
Model 1	Below median	2.86	(1.85,4.44)	<0.001	1.65	(0.84,3.25)	0.146	1.25	(0.92,1.70)	0.160
	Above median	3.72	(2.45,5.65)	<0.001	1.53	(0.77,3.05)	0.226	1.28	(0.94,1.73)	0.117
Model 2	Below median	3.01	(1.89,4.79)	<0.001	2.06	(1.01,4.18)	0.046	1.30	(0.93,1.81)	0.121
	Above median	3.77	(2.40,5.91)	<0.001	1.40	(0.68,2.89)	0.357	1.12	(0.80,1.56)	0.520
Model 3	Below median	3.04	(1.91,4.84)	<0.001	2.16	(1.06,4.40)	0.035	1.28	(0.92,1.79)	0.140
	Above median	3.77	(2.40,5.91)	<0.001	1.46	(0.71,3.01)	0.308	1.11	(0.80,1.55)	0.531
Model 4	Below median	2.99	(1.88,4.76)	<0.001	2.16	(1.06,4.42)	0.034	1.29	(0.93,1.80)	0.133
	Above median	3.74	(2.38,5.88)	<0.001	1.48	(0.71,3.05)	0.294	1.11	(0.79,1.55)	0.552
Model 5	Below median	3.01	(1.90,4.79)	<0.001	2.07	(1.02,4.20)	0.045	1.30	(0.93,1.81)	0.121

	Above median	3.71	(2.37,5.83)	<0.001	1.40	(0.68,2.89)	0.362	1.11	(0.79,1.55)	0.547
Model 6	Below median	3.02	(1.89,4.81)	<0.001	2.17	(1.06,4.44)	0.034	1.28	(0.92,1.79)	0.139
	Above median	3.61	(2.29,5.68)	<0.001	1.45	(0.70,3.01)	0.316	1.10	(0.79,1.54)	0.566
Acetaminophen burden**										
Model 1	Below median	2.99	(1.94,4.63)	<0.001	1.55	(0.78,3.10)	0.212	1.26	(0.92,1.71)	0.149
	Above median	3.59	(2.36,5.46)	<0.001	1.63	(0.83,3.21)	0.157	1.27	(0.93,1.72)	0.126
Model 2	Below median	3.18	(2.00,5.04)	<0.001	1.91	(0.93,3.93)	0.079	1.30	(0.93,1.81)	0.120
	Above median	3.59	(2.29,5.64)	<0.001	1.52	(0.75,3.08)	0.250	1.11	(0.80,1.55)	0.523
Model 3	Below median	3.21	(2.02,5.10)	<0.001	2.00	(0.97,4.14)	0.061	1.28	(0.92,1.79)	0.140
	Above median	3.59	(2.29,5.63)	<0.001	1.57	(0.77,3.20)	0.213	1.11	(0.80,1.55)	0.533
Model 4	Below median	3.15	(1.99,5.01)	<0.001	2.01	(0.97,4.16)	0.061	1.29	(0.93,1.80)	0.132
	Above median	3.57	(2.27,5.60)	<0.001	1.59	(0.78,3.25)	0.201	1.11	(0.79,1.54)	0.556
Model 5	Below median	3.19	(2.01,5.06)	<0.001	1.92	(0.93,3.95)	0.077	1.30	(0.94,1.82)	0.118
	Above median	3.53	(2.25,5.55)	<0.001	1.51	(0.74,3.07)	0.255	1.11	(0.79,1.54)	0.556
Model 6	Below median	3.20	(2.01,5.08)	<0.001	2.02	(0.97,4.18)	0.060	1.29	(0.92,1.80)	0.136
	Above median	3.42	(2.17,5.40)	<0.001	1.56	(0.76,3.20)	0.222	1.10	(0.79,1.54)	0.576

Note: NT was defined as free of any developmental disorder diagnosis; ADHD only was defined as any ADHD diagnosis without having an ASD diagnosis; ASD only was defined as any ASD diagnosis without having an ADHD diagnosis; ADHD and ASD was defined as having both ADHD and ASD diagnosis;
Model 1: Multinomial logistic regression without adjustment;
Model 2: Model 1 further adjusted for maternal age at delivery, maternal race/ethnicity, maternal education, smoking before or during pregnancy, drinking before or during pregnancy, maternal BMI, parity, child's sex, delivery type, preterm birth, and birthweight;
Model 3: Model 2 further adjusted for maternal fever during pregnancy;
Model 4: Model 2 further adjusted for maternal intrauterine infection/inflammation during pregnancy;
Model 5: Model 2 further adjusted for breastfeeding;
Model 6: Model 2 further adjusted for maternal fever, maternal intrauterine infection/inflammation during pregnancy, and breastfeeding.
* Inverse normal transformed intensity ** Sum of all the acetaminophen metabolites.
Unchanged acetaminophen: first tertile as reference; For other exposures: no detection as reference
